# Anemia in Myelofibrosis: A Focus on Proactive Management and the Role of Momelotinib

**DOI:** 10.3390/cancers16234064

**Published:** 2024-12-04

**Authors:** Haifa Kathrin Al-Ali, Andrew T. Kuykendall, Catherine E. Ellis, Janardhan Sampath, Ruben Mesa

**Affiliations:** 1 Krukenberg Cancer Center Halle, University Hospital Halle, 06120 Halle, Germany; 2 Moffit Cancer Center, Tampa, FL 33612, USA; 3 GSK plc, Collegeville, PA 19426, USAjanardhan.x.sampath@gsk.com (J.S.); 4 Atrium Health Wake Forest Baptist Comprehensive Cancer Center, Wake Forest University School of Medicine, Winston-Salem, NC 27157, USA

**Keywords:** anemia, cytopenic myelofibrosis, momelotinib, myelofibrosis, thrombocytopenia

## Abstract

**Background:** Anemia is a common and progressive clinical manifestation of myelofibrosis that may occur as part of the disease pathogenesis as well as due to the myelosuppressive effects of some treatments, with a substantial impact on quality of life, prognosis, and healthcare resource utilization. Despite these burdens, anemia management has traditionally been a secondary priority to spleen and symptom control, due in part to the limitations of available therapeutic approaches. With the initial regulatory approvals of momelotinib, a Janus kinase 1 (JAK1), JAK2, and activin A receptor type 1 inhibitor that provides anemia-related benefits in addition to addressing splenomegaly and symptoms, re-evaluation of anemia as an early and prominent treatment consideration is warranted. **Methods:** In this review, we discuss the journey of patients with myelofibrosis and anemia across various severities and clinical scenarios. **Results:** Summarized are traditional approaches to anemia management and the clinical trial efficacy and safety data that support momelotinib as an option in each setting from mild to severe anemia, including in the context of co-occurring thrombocytopenia. **Conclusions:** With the availability of momelotinib and other emerging therapies directed at anemia control, early treatment of anemia to avoid progression and support improvement in eligible patients with myelofibrosis should be a primary consideration.

## Simple Summary

Many patients with the rare blood cancer myelofibrosis have anemia (too few red blood cells), which is associated with negative effects on their general well-being, daily activities, and how long they may live. However, treating anemia in myelofibrosis has previously been thought of as less important than reducing symptoms and spleen size, in part because there were treatments available for those aspects of the disease. A newer treatment, momelotinib, is now available that can treat anemia as well as reduce spleen size and symptoms, and other treatments targeting anemia are also being evaluated in clinical trials. In light of these developments, we believe that the priority of treating anemia should be increased in myelofibrosis. In this review, we describe some different types of patients with myelofibrosis and anemia, how their treatment used to be approached, and the clinical trial data that support momelotinib as an option in those patients.

## 1. Introduction

### 1.1. Anemia in Myelofibrosis

Anemia is a prevalent and debilitating hallmark of myelofibrosis, a chronic myeloproliferative neoplasm arising de novo (primary myelofibrosis) or developing secondarily from essential thrombocythemia or polycythemia vera [1,2]. At the time of myelofibrosis diagnosis, between 30% and 40% of patients are anemic, defined by a hemoglobin level <100 g/L, and nearly all will become so over time [3,4]. In one study, 47% of patients who were not anemic at myelofibrosis diagnosis developed anemia after a median of 3.3 years [3]. In another, rates of anemia increased from 38% at myelofibrosis diagnosis to 58% at 1 year following diagnosis [4]. In addition, anemia severity over time increased with disease progression, as evidenced by a doubling in the rate of patients who were red blood cell (RBC) transfusion dependent from diagnosis to 1 year later (24% vs. 46%) [4].

The pathogenesis of anemia in myelofibrosis is multifactorial and incompletely understood, but contributions from multiple disease-related mechanisms suggest that it is an intrinsic component of myelofibrosis. These mechanisms include pro-inflammatory cytokine signaling due to aberrant Janus kinase–signal transducer and activator of transcription (JAK-STAT) pathway signaling, leading to bone marrow fibrosis and ineffective extramedullary hematopoiesis, RBC sequestration and dilution due to splenomegaly and associated increases in plasma volume, and a loss of RBCs through bleeding or destruction in circulation; dysregulated iron metabolism and non–JAK-STAT molecular alterations also contribute to dysfunctional erythropoiesis in myelofibrosis [5,6,7,8]. In addition, anemia can be induced or exacerbated by myelofibrosis treatment, as in the case of some JAK inhibitors such as ruxolitinib and fedratinib, which provide spleen and symptom benefits in many patients but are often myelosuppressive [9,10]. Notably, management considerations for anemia in myelofibrosis are distinct from those of iron, vitamin B12, and other nutrient-deficiency anemias.

### 1.2. Burden of Anemia in Myelofibrosis

The impact of anemia on patients with myelofibrosis is profound and increases as anemia severity increases. Due in part to associated symptoms such as fatigue, anemia negatively impacts health-related quality of life and daily functioning, and anemia improvement has been implicated in improved patient-reported outcomes. Post hoc clinical trial analyses have demonstrated the incremental negative effect of baseline anemia severity on physical functioning in patients with myelofibrosis; these analyses also highlighted improvements in transfusion burden and hemoglobin levels at week 24 as positively associated with multiple domains related to quality of life, mental health, and daily living [11,12].

Anemia of any severity also negatively impacts survival, with one study finding a difference of >5 years in median survival in patients with no anemia vs. those with severe anemia (hemoglobin levels <80 g/L or transfusion dependence) [13]. A hemoglobin level <100 g/L is included as a negative prognostic factor across validated myelofibrosis risk scoring systems, such as the Dynamic International Prognostic Scoring System (DIPSS) and DIPSS-plus [3,14]. Potential survival benefits with JAK inhibitors are also compromised by anemia, with an analysis of the phase 3 COMFORT studies of ruxolitinib finding that the presence of baseline anemia adversely impacted the overall survival observed with treatment [15]. Although that study found that only baseline, or disease-related, anemia was negatively prognostic, subsequent analyses from the phase 3b JUMP study illustrated that new or worsening anemia during treatment in patients who were nonanemic at baseline also negatively impacted survival in ruxolitinib-treated patients [16]. Baseline or treatment-related anemia is also associated with an increased risk of blast phase progression [17].

The quality of life and prognostic detriments of anemia also adversely affect healthcare resource utilization and costs in the management of patients with myelofibrosis. One retrospective analysis based on Medicare claims data found an incremental increase in inpatient admissions, emergency department visits, and both medical and pharmacy costs based on anemia severity, with severe anemia associated with a nearly 2-fold increase in total costs compared to mild anemia [18].

### 1.3. Reprioritizing Anemia Management

The substantial burden of anemia in myelofibrosis, and the incremental effect of increasing anemia severity on these burdens, suggests that anemia improvement, or at least the avoidance of anemia worsening, should be a key priority. However, anemia management has traditionally been secondary to spleen and symptom control in terms of treatment prioritization [19]. This may have been due in part to the aforementioned prominence of ruxolitinib in the therapeutic landscape and the absence of treatment options that directly targeted the mechanisms of anemia. Traditional approaches to anemia management include dose reduction of JAK inhibitors such as ruxolitinib to mitigate myelosuppressive effects, RBC transfusions, and supportive therapies such as erythropoiesis-stimulating agents (ESAs), androgens, and immunomodulatory drugs [19]. While these remain appropriate and potentially effective treatment options for many patients with anemia, each is associated with limitations (discussed in more detail later in this review) that may have contributed to anemia’s placement as a secondary consideration in treatment selection.

We believe this perception warrants re-evaluation in light of the emergence of newer JAK inhibitors such as momelotinib and pacritinib, as well as investigational agents that may offer anemia-related benefits. Momelotinib, a JAK1, JAK2, and activin A receptor type 1 (ACVR1) inhibitor, became the first and, to date, only treatment indicated for patients with myelofibrosis and anemia when initially approved by the US Food and Drug Administration in September 2023, with subsequent approvals by the European Commission, the UK Medicines and Healthcare products Regulatory Agency, and the Ministry of Health, Labour and Welfare of Japan [20,21,22,23]. Like all JAK inhibitors, momelotinib can improve constitutional symptoms and splenomegaly, but through its inhibition of ACVR1, it also provides anemia-related benefits [24]. As illustrated both preclinically and in a phase 2 translational biology study, ACVR1 inhibition by momelotinib decreases the production of hepcidin, a master regulator of iron metabolism, thus restoring iron homeostasis and increasing erythropoiesis [25,26]. The role of ACVR1 inhibition in anemia-related benefits in myelofibrosis is also supported by the more recent finding that pacritinib, another JAK inhibitor previously described as a JAK2/fms-related receptor tyrosine kinase 3 (FLT3)/interleukin 1 receptor-associated kinase 1 inhibitor, also inhibits ACVR1 and showed retrospective evidence of anemia-related benefits in clinical trial analyses [27,28].

### 1.4. Momelotinib

The clinical benefits of momelotinib were evaluated in three phase 3 trials, which showed spleen, symptom, and anemia benefits in the intent-to-treat (ITT) populations across trials but mixed results with respect to statistical significance (Table 1) [29,30,31]. SIMPLIFY-1 evaluated momelotinib vs. ruxolitinib in JAK inhibitor–naive patients, and the primary endpoint of noninferiority in spleen volume reduction ≥35% (SVR35) at week 24 was met; however, noninferiority was not achieved on the key secondary endpoint related to symptom improvement, as assessed by a ≥50% reduction in Total Symptom Score (TSS50) at week 24 [31]. Potential contributing factors to not achieving this secondary endpoint include a lack of stratification by TSS, leading to a higher mean TSS in the momelotinib arm; no minimum TSS required for enrollment, leading to the inclusion of patients who were asymptomatic; and a protocol design that resulted in higher rates of momelotinib vs. ruxolitinib discontinuation prior to week 24, leaving more patients with missing data to be documented as non-responders. Notably, individual symptom item improvement was comparable between the momelotinib and ruxolitinib arms [32]. 

SIMPLIFY-2 compared momelotinib vs. best available therapy (BAT), which was continued ruxolitinib in 88% of patients, in the JAK inhibitor–experienced setting. The primary endpoint of SVR35 superiority was not met, with splenic response rates notably low in both treatment arms likely due to the lack of washout of prior JAK inhibitors; however, momelotinib was nominally superior in the proportion of patients achieving TSS50, a key secondary endpoint [30]. A second trial in the JAK inhibitor–experienced setting, MOMENTUM, was conducted to more definitively characterize the benefits of momelotinib and so was restricted to patients who were symptomatic (TSS ≥ 10) and anemic (hemoglobin level <100 g/L) at baseline and included danazol, an androgen commonly used to manage myelofibrosis-related anemia, as the comparator; momelotinib was superior for both the primary TSS50 and key secondary SVR35 endpoints [29]. In all three trials, momelotinib demonstrated an improvement in anemia, which was assessed as a key secondary endpoint via a stringent definition of transfusion independence response at week 24 that required no transfusions and all hemoglobin levels ≥80 g/L in the previous 12 weeks [29,30,31]. Other secondary and exploratory measures of anemia improvement across the phase 3 program, including the mean hemoglobin levels over time, the rate of transfusions through week 24, and the zero transfusion rate, also favored momelotinib [29,30,31].

**Figure 1:**
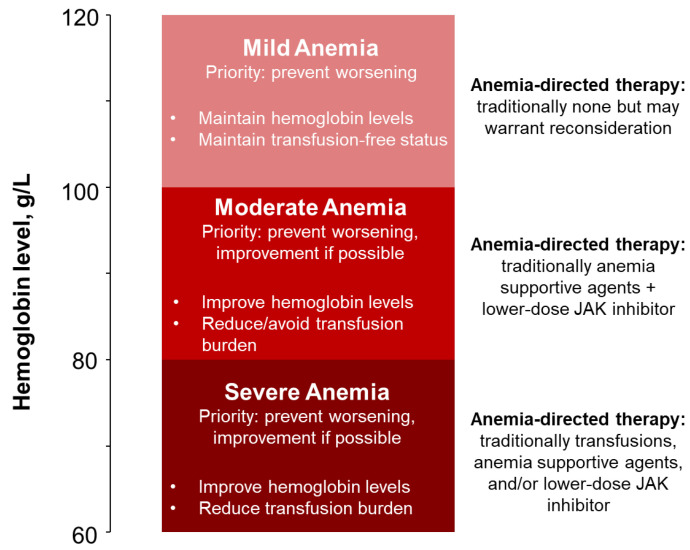
Anemia management priorities and traditional approaches across the patient journey in myelofibrosis. JAK, Janus kinase.

Given the substantial burden of anemia in myelofibrosis, the approval of a treatment such as momelotinib, with robust clinical trial evidence of anemia, spleen, and symptom benefits, should now facilitate the elevation of proactive anemia management to a key consideration in eligible patients (Figure 1). However, the breadth and complexity of the momelotinib clinical trial data, much of which were not limited to patients with anemia, may make it challenging to identify those patients in practice for whom anemia management can and should be prioritized. To highlight the importance of early treatment of anemia as a paradigm shift in myelofibrosis management, we review several clinical scenarios that may be encountered throughout the journey of a patient with myelofibrosis and anemia, describing the rationale for early treatment, the limitations of previously available approaches to treatment, and the clinical trial evidence that supports momelotinib as an additional option for proactive anemia management in both the JAK inhibitor–naive and –experienced settings.

## 2. Mild Anemia

We first consider the case of Patient A, a 77-year-old man who previously presented with palpable splenomegaly, constitutional symptoms including weight loss and night sweats, and a hemoglobin level of 109 g/L. Following a bone marrow biopsy, a diagnosis of myelofibrosis was confirmed, and he has returned to you for initial treatment. Subsequent laboratory testing revealed that the hemoglobin level has decreased slightly to 102 g/L, but the patient does not report any substantial anemia-associated symptoms such as fatigue. Other laboratory findings are unremarkable (Table 2).

As anemia progresses over time with advancing disease course [4], Patient A represents the most likely scenario in which mild anemia will be encountered: a newly diagnosed patient who is JAK inhibitor naive. An initial challenge in the evaluation of this patient is that the very definition of mild anemia can be variable; according to the World Health Organization (WHO), the upper threshold that broadly defines anemia is any hemoglobin level that is below the lower limit of normal, which varies by factors such as age, sex (e.g., 120 g/L in women vs. 130 g/L in men), race, and geography [13,39,40]. Per these WHO guidelines, a lower threshold of 110 g/L defines mild anemia [39]. However, this is contradicted by other sources, such as the Common Terminology Criteria for Adverse Events (CTCAE) Version 5.0, which employ a threshold of 100 g/L to define grade 1 anemia; the clinical trial data included in momelotinib regulatory labeling also favor this lower threshold of 100 g/L [20,23,41].

In a transplant-ineligible patient with mild anemia who also presents with spleen and symptom burdens, prioritization of anemia management is also challenged by the fact that such patients have not traditionally been considered for anemia-directed therapy (Figure 1) [42]. However, even mild anemia may have clinical consequences, with one study finding that median survival was reduced by 3 years in patients with mild anemia (hemoglobin levels ≥100 g/L to below the sex-adjusted lower limit of normal) vs. no anemia [13,39]. Thus, there is strong rationale for considering some patients with mild anemia for treatment, particularly if therapeutic options that can also provide spleen and symptom benefits are available. Factors such as comorbidities, the burden of anemia-related symptoms (e.g., fatigue), and the stability of hemoglobin levels over time (e.g., if a patient’s hemoglobin level is 102 g/L, but this represents a substantial decrease from the previous reading and other causes have been ruled out) may predict patients who are more likely to experience worsening anemia and may benefit from earlier anemia management, although these are not well defined.

### 2.1. Previous Treatment Options in Mild Anemia

We pause here to acknowledge that hematopoietic stem cell transplant remains the only curative treatment option in myelofibrosis and should always be an initial consideration, dependent on prognostic risk scoring in line with the latest guideline recommendations and factors such as age and comorbidity, which might impact eligibility [43]. As considerations for transplant have been reviewed elsewhere [43,44,45], we focus on treatment decisions in patients with myelofibrosis and anemia for whom transplant is not an option.

JAK inhibitors are the traditional treatment of choice in this patient scenario, and selection may be informed by the potential impact on this patient’s mild anemia. In the phase 3 trials of both the JAK1/JAK2 inhibitor ruxolitinib and JAK2/FLT3 inhibitor fedratinib, indicators of anemia worsening such as decreased mean hemoglobin levels and increased transfusions were evident in the initial weeks of treatment [46,47,48]. Dose reductions of these JAK inhibitors may be required in the event of new or worsening anemia [49,50], which can compromise clinical efficacy [10,51]; furthermore, the Response to Ruxolitinib After 6 Months (RR6) prognostic model identifies both lower dosing and RBC transfusion need among the risk factors associated with poor survival after 6 months of ruxolitinib [51]. In addition to dose reductions of ruxolitinib or fedratinib, the introduction of concomitant anemia supportive therapies such as ESAs may also be considered but are more frequently reserved for patients with greater anemia severity and thus will be discussed later in this review [2,42]. In the United States, pacritinib is also an option and has demonstrated anemia-related benefits in retrospective analyses from the phase 3 PERSIST-2 trial [28]. However, as of writing this review, pacritinib is indicated specifically in patients with severe thrombocytopenia and is therefore only an option if the patient with mild anemia also has platelet counts <50 × 10^9^/L [27]. Considerations for the treatment of patients with both anemia and thrombocytopenia are discussed in more detail later in this review.

### 2.2. Momelotinib Clinical Trial Evidence in Mild Anemia

Clinical evidence for momelotinib in patients with mild anemia comes primarily from a subgroup analysis of SIMPLIFY-1, which defined this population by baseline hemoglobin levels ≥ 100 to <120 g/L [33]. The majority of these patients were transfusion independent at baseline (90% in the momelotinib arm and 83% in the ruxolitinib arm). Most patients retained this status at week 24 with momelotinib (86%), while 58% did so with ruxolitinib, which suggests that momelotinib was associated with higher maintenance of transfusion independence and the prevention of anemia progression. Among the 19 patients who were not transfusion independent at baseline, 29% (2 of 7) became so at week 24 with momelotinib vs. 17% (2 of 12) with ruxolitinib [33].

Beyond this stringent response/non-response endpoint, which includes data only from the 12 weeks before week 24 and does not capture anemia-related benefits that may be meaningful to patients but fall below the threshold for response, longitudinal analyses of transfusion burden over time (units per 28 days) have also been reported for this subgroup. Compared with baseline, 93% of patients had stable or reduced transfusion intensity throughout the 24 weeks of momelotinib treatment vs. 51% with ruxolitinib [33]. Again, demonstrating the association of momelotinib with the avoidance of anemia worsening, 94% of patients who were transfusion free at baseline retained this status throughout treatment vs. only 50% with ruxolitinib [33]. Mean hemoglobin levels also indicate the avoidance of anemia worsening with momelotinib and remained stable over time. In contrast, mean hemoglobin levels decreased with ruxolitinib and plateaued below the 100-g/L threshold; notably, mean hemoglobin levels increased in patients initially randomized to ruxolitinib who switched to open-label momelotinib after week 24 [33].

SVR35 (26% vs. 25%) and TSS50 (35% vs. 45%) results in this subgroup were consistent with the ITT; notably, despite the numerically higher TSS50 rate with ruxolitinib, individual symptom items were stable or improved in a similar number of patients between arms [33]. Moreover, dual responses (SVR35 or TSS50 + transfusion independence) were also common with momelotinib; all (19 of 19 [100%]) and nearly all (24 of 25 [96%]) splenic and symptom responders, respectively, were also transfusion independent at week 24, compared with 8 of 17 (47%) and 17 of 29 (59%) with ruxolitinib [33]. Thus, while JAK inhibitors such as ruxolitinib remain an appropriate choice for Patient A, clinical trial evidence also supports the consideration of momelotinib in such a patient with mild anemia as well as spleen and symptom burdens to comprehensively address the clinical manifestations of myelofibrosis by preventing anemia worsening and providing spleen and symptom control.

## 3. Moderate Anemia

Continuing the patient journey with increasing anemia severity, we now consider some patients who have progressed beyond the early stages of anemia to the point where they would be traditionally considered for anemia-directed therapy (Figure 1) [42]. Patient B, a 63-year-old woman, is similar to Patient A in that she presents with recently diagnosed myelofibrosis, palpable splenomegaly, and constitutional symptoms; however, Patient B also reports worsening fatigue, and, on laboratory workup, her hemoglobin level is found to be 91 g/L. Although transplant would be an appropriate initial therapeutic option for this patient, she is not interested at this time and would prefer to pursue other treatment options (Table 2). Meanwhile, Patient C, a 72-year-old man with a history of interstitial lung disease, has been receiving JAK inhibitor treatment for myelofibrosis under your care for 2 years and is generally happy with the spleen and symptom control he is experiencing. However, Patient C’s hemoglobin levels have prompted transfusion twice in the past 2 months, and his most recent laboratory workup shows worsening anemia, with a hemoglobin level decrease from 98 g/L at the previous assessment to 87 g/L at present (Table 2).

As with mild anemia, the definition of moderate anemia that is applied will inform treatment considerations for both Patient B and Patient C. WHO and CTCAE grading are aligned with a threshold of 80 g/L defining severe anemia; thus, moderate anemia can be considered to constitute hemoglobin levels from 80 to <110 (WHO) or 80 to <100 (CTCAE) g/L [39,41]. These thresholds are consistent with updated International Working Group-European LeukemiaNet criteria for anemia response, which broadly suggest definitions of baseline anemia in the context of clinical trials, considering thresholds <110 g/L for men and <100 g/L for women [52]. It is in these patients with moderate anemia that anemia-directed treatment is traditionally first considered, typically in the form of supportive therapies [53]. While transfusion dependence is generally associated with more severe anemia (and thus will be discussed in more detail later in this review), occasional transfusions may be introduced as part of the management approach at this stage. Therefore, the avoidance of transfusion dependence and decreasing transfusion intensity become greater priorities in these patients, along with improvement in hemoglobin levels to reduce anemia severity if possible.

### 3.1. Previous Treatment Options in Moderate Anemia

Traditional anemia supportive therapies include ESAs, androgens such as danazol, and immunomodulatory drugs with or without corticosteroids [2]. None of these agents are specifically indicated for the management of anemia in myelofibrosis, they do not directly target its mechanism, and high-quality evidence of their utility is limited [2]. ESAs, such as epoetin or darbepoetin alfa, may be useful in patients with serum erythropoietin levels <125 IU/L. Smaller spleen size and transfusion independence are also associated with an increased likelihood of response to ESAs; however, even in these situations, responses are limited in number and duration [2,54,55]. Among the androgens, danazol has the most evidence in patients with myelofibrosis and is generally associated with lower toxicity [2,56,57]. However, screening for prostate cancer or elevated prostate-specific antigen levels and liver function tests are recommended [2,56,57]. Response rates are low, particularly in patients with transfusion dependence, and the onset of response may be slow [2,56,57]. Response rates are also low with immunomodulatory drugs such as thalidomide or lenalidomide, and toxicity is a concern albeit somewhat mitigated when used in combination with prednisone [2,58]. Notably, investigational agents with anemia indications in therapeutic areas outside myelofibrosis, such as luspatercept, are also often used off label or in the context of clinical trials [9,59]. The ongoing phase 3 INDEPENDENCE trial of luspatercept in combination with a JAK2 inhibitor will shed additional light on this approach to anemia management in transfusion-dependent patients with myelofibrosis [60].

In a transplant-ineligible patient with spleen and symptom burdens in addition to moderate anemia, these anemia supportive therapies would traditionally be paired with a JAK inhibitor. In the case of Patient B, who is JAK inhibitor naive with moderate anemia, considerations for JAK inhibitor selection are largely the same as those described for Patient A, who is also JAK inhibitor–naive but with mild anemia. However, increased regard for how the JAK inhibitor selected might compound anemia burden may be warranted in Patient B. For a patient like Patient C, who is experiencing new or worsening anemia on a first-line JAK inhibitor such as ruxolitinib, it may be possible to continue his current JAK inhibitor, typically at a reduced dose to mitigate its myelosuppressive effects, and add an anemia supportive agent; however, we explore later in this review how this approach may not be optimal for all patients. Switching JAK inhibitors is also an option, particularly to pacritinib or momelotinib, which may offer anemia-related benefits, although concerns related to ruxolitinib discontinuation syndrome (i.e., loss of spleen and symptom response, hemodynamic decompensation) must be taken into account [61,62].

### 3.2. Momelotinib Clinical Trial Evidence in Moderate Anemia

#### 3.2.1. Moderate Anemia: JAK Inhibitor Naive

There is ample clinical trial evidence surrounding momelotinib in both the JAK inhibitor–naive and –experienced settings in the context of moderate anemia. In the JAK inhibitor–naive setting, this evidence comes from a subgroup analysis of SIMPLIFY-1, which considered patients with hemoglobin levels <100 g/L; thus, patients with more severe anemia were also included in this dataset [33]. This subgroup comprised 86 patients in the momelotinib arm (58 with moderate anemia) and 94 patients in the ruxolitinib arm (73 with moderate anemia), most of whom (63%) had some transfusion burden at baseline [33]. In patients who were transfusion independent at baseline, 72% vs. 34% maintained this status at week 24 with momelotinib vs. ruxolitinib, while an additional 36% vs. 21% who were not transfusion independent at baseline became so at week 24, demonstrating evidence of both the avoidance of anemia worsening and anemia-related improvement with momelotinib [33].

In longitudinal analyses of transfusion intensity, 84% on momelotinib vs. 43% on ruxolitinib had stable or reduced transfusion intensity during treatment, despite the fact that fewer patients started out as transfusion free at baseline before treatment with momelotinib (34%) vs. ruxolitinib (53%); 86% vs. 30% of those patients who were transfusion free at baseline remained so during treatment with momelotinib vs. ruxolitinib, while 25% vs. 11% of those with some transfusion burden at baseline also became transfusion free during treatment [33]. Mean hemoglobin levels improved rapidly with momelotinib but declined before plateauing with ruxolitinib [33]. These increases in anemia worsening with ruxolitinib were observed despite mean daily doses decreasing over time, indicative of dose reductions due to cytopenias; in contrast, momelotinib was administered at near-full dose through week 24 [33]. SVR35 (31% vs. 33%) and TSS50 (25% vs. 35%) for momelotinib vs. ruxolitinib were consistent with the ITT and the mildly anemic subgroup [33]. With respect to dual responses, 23 of 27 (85%) splenic responders and 15 of 21 (71%) symptom responders in the momelotinib arm were also transfusion independent at week 24, compared with 7 of 31 (23%) and 9 of 33 (27%) with ruxolitinib [33]. Thus, if treatment of anemia, splenomegaly, and symptoms were prioritized equally, the clinical trial evidence would support the use of momelotinib in JAK inhibitor–naive patients with moderate anemia and spleen/symptom burdens to maintain or improve anemia-related burdens.

#### 3.2.2. Moderate Anemia: JAK Inhibitor Experienced

In the JAK inhibitor–experienced setting, evidence of momelotinib efficacy and safety following previous ruxolitinib treatment also comes from SIMPLIFY-1, although data are not available specifically from the moderate to severely anemic subpopulation. Although SIMPLIFY-1 enrolled JAK inhibitor–naive patients at baseline, after week 24, all patients received open-label momelotinib, meaning that patients initially randomized to ruxolitinib were immediately transitioned to momelotinib with no washout [37]. Mean hemoglobin levels improved rapidly after crossover to momelotinib, and mean spleen volume was maintained; 46% of patients who were not transfusion independent at crossover became so by week 12 [37]. Although 57% of patients required a dose modification of ruxolitinib through week 24, 90% initiated momelotinib at the full 200-mg daily dose at week 24, and most maintained this dosing 12 weeks after crossover [37]. No ruxolitinib discontinuation syndrome or unexpected safety signals were observed after crossover [37]. Thus, patients can be safely transitioned to momelotinib from ruxolitinib without compromising efficacy [37].

JAK inhibitor–experienced data specifically in patients with moderate to severe anemia comes from MOMENTUM, in which all patients had hemoglobin levels <100 g/L at baseline; approximately half of the patients were also transfusion dependent [29]. As summarized in Table 1, momelotinib exhibited significantly improved spleen, symptom, and transfusion independence rates vs. danazol in this patient population [29]. Looking more closely at anemia-related benefits in this study, longitudinal analysis of transfusion intensity found that while only 20% and 17% of patients in the momelotinib and danazol arms were transfusion free at baseline, 92% retained this status on momelotinib treatment vs. only 64% with danazol, while 21% vs. 7% of those with transfusion burden at baseline also became transfusion free during treatment; 48% vs. 38% of patients overall had improved transfusion intensity [36]. Thus, switching to momelotinib in moderately anemic patients who require anemia-directed therapy is associated with anemia-related benefits and continued spleen and symptom control; patients with lower transfusion burden maintain this status and avoid anemia worsening.

## 4. Severe Anemia/Transfusion Dependence

Hemoglobin levels <80 g/L and/or transfusion dependence are consistently defined as severe anemia; by the time a patient has reached this stage, the anemia burden is substantial, and most must rely on frequent transfusions to temporarily improve or maintain hemoglobin levels [39,41]. While severe anemia is often encountered in the context of more advanced disease when a patient is likely to be JAK inhibitor experienced, it is important to note that some patients may present with severe anemia and require transfusions even in the JAK inhibitor–naive setting.

Consider the case of Patient D, a 73-year-old man with a history of congestive heart failure. This patient was diagnosed with myelofibrosis last month and has palpable splenomegaly, pronounced fatigue, and a laboratory workup that revealed several abnormalities, including a hemoglobin level of 69 g/L. He was immediately referred for a transfusion and now needs to begin treatment (Table 2). On the other hand, Patient E is a 68-year-old woman who has been under your care for myelofibrosis for the past 3 years. Transplant was previously discussed with the patient as an appropriate therapeutic option, but she declined due to personal preference and has been doing well on her initial JAK inhibitor since then. However, she has recently become increasingly dependent on transfusions to maintain her hemoglobin levels, receiving three in the past month; nevertheless, her latest laboratory workup revealed a hemoglobin level of 82 g/L (Table 2).

### 4.1. Previous Treatment Options in Severe Anemia

Anemia management is naturally a priority in both of these patients with severe anemia, but while transfusions may temporarily relieve anemia-related symptoms, they also compound the quality of life, survival, and healthcare resource burdens that these patients will experience (Figure 1). Post hoc clinical trial analyses have demonstrated that patients who are transfusion dependent at baseline score worse vs. those who are transfusion requiring (infrequent transfusions) or independent across patient-reported outcome domains related to quality of life; physical, social, and mental functioning; and daily living [11]. Transfusion dependence at diagnosis, as seen in Patient D, is also associated with poor overall survival, with one study observing median survival of only 2.6 years compared with 8 years in patients who were transfusion independent [63]. Becoming transfusion dependent at any time, as seen in Patient E, is also associated with poor overall survival and is included as an independent negative prognostic factor beyond anemia itself in DIPSS-plus, providing additional rationale to prioritize the maintenance of transfusion independence whenever possible [14,63]. Conversely, a consistent association between achieving transfusion independence at week 24 with momelotinib and prolonged overall survival has also been reported [64,65]. Similar to the effects observed with anemia severity, multiple claims-based analyses have demonstrated an incremental increase in costs and healthcare resource utilization with increasing transfusion burden, with one analysis identifying total medical costs up to nine times higher for transfusion-dependent patients than for those who were not [18,66]. Additionally, chronic transfusion dependence can lead to high rates of complications, including iron overload, which can damage the liver, heart, and other organs, and an increased risk of infections [67,68,69].

Eligible patients with severe anemia and transfusion dependence will likely have intermediate- or high-risk disease and should routinely be considered for transplant [43]. Otherwise, JAK inhibitors are the primary treatment option for a severely anemic, transplant-ineligible patient with spleen and symptom burden. For JAK inhibitor–naive patients with severe anemia like Patient D, the selection of an initial JAK inhibitor that will not compound their anemia burden is even more critical than in those previously discussed. For severely anemic patients like Patient E who are already receiving a JAK inhibitor, considerations are largely the same as in a patient with moderate anemia, although transfusion dependence is associated with a decreased likelihood of response to many traditional anemia supportive therapies. Thus, switching to a JAK inhibitor that may be able to offer anemia-related benefits should be a prominent consideration.

### 4.2. Momelotinib Clinical Trial Evidence in Severe Anemia

#### 4.2.1. Severe Anemia: JAK Inhibitor Naive

Momelotinib has been extensively studied in both the JAK inhibitor–naive and –experienced settings in the context of severe anemia and transfusion dependence. In a phase 2 translational biology study, 88% of patients were JAK inhibitor naive, yet all patients evaluable for transfusion status (98%) were transfusion dependent at baseline, providing key evidence for the often overlooked JAK inhibitor–naive population with a heavy transfusion burden [26]. While 34% of patients achieved the strict criteria for transfusion independence at week 24, additional insights are gleaned from longitudinal analyses of transfusion intensity [26]; 85% of patients had a numeric reduction in transfusion intensity with treatment [36]. While no patients were transfusion free at baseline, 22% became transfusion free with treatment, and 90% of patients overall had improved or stable transfusion intensity [36]. This trial therefore provides robust evidence that momelotinib can provide anemia-related benefits in many JAK inhibitor–naive patients with severe anemia.

While we have already reviewed the results in the SIMPLIFY-1 subgroup with hemoglobin levels <100 g/L, including those who were transfusion dependent at baseline, in the context of moderate anemia, separate results have also been reported in the subgroup of patients with hemoglobin levels <80 g/L [33]. All of these patients were transfusion dependent at baseline, but 29% vs. 14% experienced anemia improvement with momelotinib vs. ruxolitinib to the point where they achieved transfusion independence at week 24. In longitudinal analyses of transfusion intensity, 79% with momelotinib vs. 38% with ruxolitinib had stable or reduced transfusion intensity with treatment, and nearly twice as many patients were transfusion free (18% vs. 10%) for the duration of treatment [33].

#### 4.2.2. Severe Anemia: JAK Inhibitor Experienced

In the JAK inhibitor–experienced setting, previously discussed evidence regarding transition from ruxolitinib to momelotinib in SIMPLIFY-1, as well as the MOMENTUM trial in patients with hemoglobin levels <100 g/L, also applies to patients with severe anemia [29,37]. In addition, a subgroup analysis of the SIMPLIFY-2 trial focused on patients who were non-transfusion independent at baseline has also been described; because there was no washout of prior therapy in this trial, and 88% of patients in the BAT arm continued on ruxolitinib, this population effectively represents those who, in practice, may continue on a reduced-dose first-line JAK inhibitor and add an anemia supportive agent vs. switching to a different JAK inhibitor [38]. The analysis suggests that switching to momelotinib in this context may be advantageous, as week 24 transfusion independence (35% vs. 3%), SVR35 (10% vs. 3%), and TSS50 (29% vs. 0%) all favored momelotinib; these rates are comparable to those observed in the ITT for momelotinib, but lower for BAT [38]. Moreover, 4 of 7 splenic responders (57%) and 10 of 21 symptom responders (48%) who were not transfusion independent at baseline achieved transfusion independence with momelotinib at week 24; no patients achieved such dual responses with BAT. Mean hemoglobin levels also increased over time with momelotinib compared with BAT, despite a lower rate of transfusions through week 24 [38].

While momelotinib was initiated at the full 200 mg daily dose in all patients in this subgroup, 59% (17 of 29) who received ruxolitinib in the BAT arm initiated ruxolitinib at ≤10 mg twice daily; nevertheless, mean dose intensity was maintained in the momelotinib arm over time, while the percentage of patients receiving lower-dose ruxolitinib continued to increase through week 24 [38]. Notably, ESAs were the most common anemia supportive therapies administered in the BAT arm, with four of five patients receiving an ESA in combination with ruxolitinib; none of these patients in the subgroup of interest achieved a spleen, symptom, or transfusion independence response at week 24, further suggesting that this strategy may not be an optimal approach in patients with a baseline transfusion burden [38]. Collectively, these datasets highlight momelotinib as an effective treatment option for spleen, symptom, and anemia-related benefits even in the context of severe anemia and transfusion dependence, a setting in which anemia management should already be a top priority.

## 5. Additional Considerations

Having reviewed the patient journey from mild through severe anemia, it is important to note that there are some factors that warrant consideration across these different settings.

### 5.1. Safety

Given the substantial quality-of-life impact of anemia and transfusions, an individualized approach to anemia management is required that considers patient characteristics and preferences. The presence of certain comorbidities (e.g., renal or hepatic impairment) in any of the patients previously discussed may inform treatment selection or switching, and the tolerability of JAK inhibitors or other therapies under consideration will also impact these decisions [23,27,49,50]. In the case of momelotinib, monitoring for adverse events consistent with the JAK inhibitor therapeutic class, including infections, hepatotoxicity, major adverse cardiovascular events, thrombosis, and malignancies, is recommended; however, momelotinib is initiated at the full 200-mg daily dose regardless of baseline cytopenias, renal impairment, or mild-to-moderate hepatic impairment [23].

The long-term safety profile of momelotinib was thoroughly characterized in a pooled analysis of data from the phase 3 clinical trial program [35]. Across 725 patients, the median duration of exposure was 11.3 months, and the relative dose intensity was maintained at a median of 97.3% during that time [35]. The most common nonhematologic adverse events were diarrhea (27%) and nausea (19%), and most were low grade, which is notable as no protocol-mandated prophylaxis was required [35]. Peripheral sensory neuropathy, an early safety signal in previous trials of momelotinib, was reported in 12% of patients and was not considered a reaction of significant concern [35]. Other adverse events of interest consistent with the JAK inhibitor therapeutic class, including infections, cytopenias, major adverse cardiovascular events, and nonmelanoma skin cancer, were consistent with previous observations and did not increase in frequency over time; no cumulative toxicity was noted [35]. In the context of patients with anemia, the SIMPLIFY-1 subgroup analyses in patients with mild, moderate, and severe anemia notably did not identify any new safety signals in these populations [33]. This safety profile of momelotinib should be considered in the context of a given patient’s comorbidities and managed in accordance with the regulatory label [20,23].

### 5.2. Thrombocytopenia

A key consideration in patients with myelofibrosis and anemia is the potential for co-occurring thrombocytopenia, which is characteristic of the so-called myelodepletive phenotype associated with an increased frequency of high-molecular-risk mutations and poor outcomes [70]. In one study, 18% of patients presented with moderate or severe thrombocytopenia (platelet counts <100 × 10^9^/L) at diagnosis and, as with anemia, incidence increased over time [4]. Patients with severe thrombocytopenia are more likely to be anemic and transfusion dependent, with poor overall survival and significant rates of leukemic transformation [71]. Moderate or severe thrombocytopenia also joins anemia and transfusion dependence as a negative prognostic indicator in myelofibrosis risk stratification (DIPSS-plus) [14].

#### 5.2.1. Previous Treatment Options for Patients with Thrombocytopenia

For any of the previously discussed patients, how might the approach to treatment have changed if, in addition to hemoglobin levels indicative of anemia, their laboratory findings also indicated thrombocytopenia? When considering JAK inhibitor selection in a patient with both anemia and thrombocytopenia, note that ruxolitinib has mandated starting dose reductions based on baseline platelet counts, while both ruxolitinib and fedratinib are dose-reduced in the event of decreasing platelet counts upon treatment and are not indicated in patients with counts <50 × 10^9^/L [49,50]. Given the potential impact of dose reduction on clinical efficacy [10,51], JAK inhibitors that can be administered at the full daily dose may be preferred. In that context, pacritinib has received accelerated approval in the United States based on spleen volume reduction specifically in patients with platelet counts <50 × 10^9^/L; given the retrospective evidence of anemia benefits in pacritinib-treated patients, consideration should be given in eligible patients with anemia [27,28]. However, pacritinib’s continued approval in the United States and availability in other regions will depend on the outcome of the phase 3 PACIFICA trial, expected to read out in 2026 [72].

#### 5.2.2. Momelotinib in Patients with Thrombocytopenia

Although dose reduction for decreased platelet counts during treatment is also required with momelotinib, patients with platelet counts <50 × 10^9^/L are eligible to initiate treatment; the starting dose is 200 mg daily regardless of baseline platelet counts, and there are no prospective data suggesting that initiation at a lower dose is appropriate or warranted in patients with counts <50 × 10^9^/L [20,23]. All three phase 3 trials of momelotinib included patients with moderate thrombocytopenia (platelet counts 50–100 × 10^9^/L), while SIMPLIFY-2 and MOMENTUM also enrolled some patients with severe thrombocytopenia (<50 × 10^9^/L) [34].

A post hoc analysis of momelotinib efficacy and safety in the populations with platelet counts <100 × 10^9^/L in each trial found SVR35, TSS50, and transfusion independence rates comparable to or higher than those observed in the ITT, including patients who were both thrombocytopenic and anemic (hemoglobin levels <100 g/L). In contrast, the results from the SIMPLIFY studies found lower response rates with ruxolitinib in thrombocytopenic patients compared with the ITT, perhaps due in part to associated dose reductions [34]. Momelotinib dose intensity remained high throughout treatment, with mean daily doses >150 mg across all three trials [34]. In momelotinib-treated patients, <20% of patients had dose reductions and <10% of patients discontinued treatment due to thrombocytopenia, and the rates of safety signals of potential concern in this subpopulation such as bleeding and hemorrhage were low [34]. Mean platelet counts remained stable over time in the overall trial populations as well as in the anemic and thrombocytopenic subgroups [33,34,35]. Although the number of patients with platelet counts <50 × 10^9^/L in these trials was small, the results were generally consistent with those from the <100 × 10^9^/L group [34]. Overall, robust clinical trial evidence supports the use of momelotinib in patients with myelofibrosis and thrombocytopenia. 

## 6. Conclusions

Anemia and transfusions impose a substantial burden on patients with myelofibrosis, providing strong rationale for prioritizing the avoidance of anemia worsening and/or transfusion dependence and the achievement of anemia-related benefits as treatment goals. With JAK inhibitors being the mainstay of myelofibrosis therapy in eligible patients who also have spleen and symptom burden, the selection of an optimal JAK inhibitor in the context of anemia burden is of paramount importance. While traditionally this consideration was deprioritized given the lack of available treatment options beyond transfusions and supportive therapy, the approval of momelotinib and the many emerging therapies under evaluation for anemia-related benefits should bring anemia management to the forefront.

Anemia-related factors such as hemoglobin level and transfusion status may help to inform the treatment of patients with myelofibrosis who have anemia. However, many additional patient-specific considerations may inform therapeutic decision-making, including the need for symptom and spleen control, prior JAK inhibitor exposure, quality-of-life impacts, and comorbidities such as thrombocytopenia. This review summarizes the clinical trial evidence supporting momelotinib as an appropriate treatment option for patients with myelofibrosis and anemia in the context of five hypothetical patients representing different scenarios that may be encountered in practice. The evidence supports early treatment with momelotinib to avoid transfusion dependence when possible, and that switching to momelotinib in patients who develop anemia on other JAK inhibitors may provide greater benefits than dose reductions and the addition of anemia supportive therapy. Future real-world analyses may provide insights into the use of momelotinib in scenarios not described here (e.g., patients with cytopenic myelofibrosis who have anemia but not necessarily symptom burden); early real-world reports on momelotinib use in clinical practice are consistent with the clinical trial results discussed here regarding its anemia-related benefits [73,74]. While longitudinal analyses of transfusion burden in clinical trials suggest that most patients derive some anemia-related benefit with momelotinib, these benefits are not universal. Therefore, future studies may also explore whether combination therapy with momelotinib and investigational anemia-directed agents has the possibility to lead to deeper responses in more patients; momelotinib may be well suited as a combination partner given its spleen, symptom, and anemia benefits as well as safety profile. Ultimately, with the availability of momelotinib and future potential anemia-directed therapies, treating anemia early should now become a priority in patients with myelofibrosis.

## Figures and Tables

**Table 1:** Summary of momelotinib phase 3 trials (intent-to-treat populations) [29,30,31].

	SIMPLIFY-1		SIMPLIFY-2		MOMENTUM	
	JAK Inhibitor Naive		JAK Inhibitor Experienced		JAK Inhibitor Experienced, Symptomatic and Anemic	
	Momelotinib (n = 215)	Ruxolitinib (n = 217)	Momelotinib (n = 104)	BAT (n = 52)	Momelotinib (n = 130)	Danazol (n = 65)
**SVR35**	27%	29%	7%	6%	22%	3%
Endpoint	Primary (noninferiority)		Primary (superiority)		Key secondary (superiority)	
Significance	*p* = 0.011		*p* = 0.90		*p* = 0.0011	
**TSS50**	28%	42%	26%	6%	25%	9%
Endpoint	Key secondary (noninferiority)		Key secondary (superiority)		Primary (superiority)	
Significance	*p* = 0.98		Nominal *p* = 0.0006		*p* = 0.0095	
**TI**	67%	49%	43%	21%	30%	20%
Endpoint	Key secondary (superiority)		Key secondary (superiority)		Key secondary (noninferiority)	
Significance	Nominal *p* < 0.001		Nominal *p* = 0.0012		*p* = 0.0116	

BAT, best available therapy; JAK, Janus kinase; SVR35, spleen volume reduction ≥ 35%; TI, transfusion independent; TSS50, Total Symptom Score reduction ≥ 50%.

**Table 2:** Clinical scenarios in patients with myelofibrosis and anemia ^a^.

	Sex	Age, Years	JAK Inhibitor Status	Transplant Eligibility?	Hb Level, g/L	Spleen/ Symptom Burden?	Transfusion Status	Relevant Momelotinib Publications
**Mild anemia**								
Patient A	Male	77	Naive	No (age)	102	Yes	TI	Efficacy/safety: [33] Thrombocytopenia: [34] Long-term safety: [35]
**Moderate anemia**								
Patient B	Female	63	Naive	Yes (but deferred due to patient preference)	91	Yes	TI	Efficacy/safety: [33] Thrombocytopenia: [34] Long-term safety: [35]
Patient C	Male	72	Experienced	No (pulmonary comorbidity)	87	Yes	TR (1 unit/month)	Efficacy/safety: [29,36] Switching: [37,38] Thrombocytopenia: [34] Long-term safety: [35]
**Severe anemia**								
Patient D	Male	73	Naive	No (cardiovascular comorbidity)	69	Yes	TR (1 unit/month)	Efficacy/safety: [26,33,36] Thrombocytopenia: [34] Long-term safety: [35]
Patient E	Female	68	Experienced	Yes (but deferred due to patient preference)	82	Yes	TD (3 units/month)	Efficacy/safety: [29,36] Switching: [37,38] Thrombocytopenia: [34] Long-term safety: [35]

Hb, hemoglobin; JAK, Janus kinase; TD, transfusion dependent; TI, transfusion independent; TR, transfusion requiring. ^a^ Patients are hypothetical and representative of potential presentations of myelofibrosis and anemia that may be encountered in practice.
